# Lung Cancer Diagnosed Through Screening, Lung Nodule, and Neither Program: A Prospective Observational Study of the Detecting Early Lung Cancer (DELUGE) in the Mississippi Delta Cohort

**DOI:** 10.1200/JCO.21.02496

**Published:** 2022-03-08

**Authors:** Raymond U. Osarogiagbon, Wei Liao, Nicholas R. Faris, Meghan Meadows-Taylor, Carrie Fehnel, Jordan Lane, Sara C. Williams, Anita A. Patel, Olawale A. Akinbobola, Alicia Pacheco, Amanda Epperson, Joy Luttrell, Denise McCoy, Laura McHugh, Raymond Signore, Anna M. Bishop, Keith Tonkin, Robert Optican, Jeffrey Wright, Todd Robbins, Meredith A. Ray, Matthew P. Smeltzer

**Affiliations:** ^1^ Multidisciplinary Thoracic Oncology Program, Baptist Cancer Center, Memphis, TN; ^2^ Mid-South Imaging and Therapeutics, Memphis, TN; ^3^ Memphis Lung Physicians, Memphis, TN; ^4^ Division of Epidemiology, Biostatistics, and Environmental Health, School of Public Health, University of Memphis, Memphis, TN

## Abstract

**PURPOSE:**

Lung cancer screening saves lives, but implementation is challenging. We evaluated two approaches to early lung cancer detection—low-dose computed tomography screening (LDCT) and program-based management of incidentally detected lung nodules.

**METHODS:**

A prospective observational study enrolled patients in the early detection programs. For context, we compared them with patients managed in a Multidisciplinary Care Program. We compared clinical stage distribution, surgical resection rates, 3- and 5-year survival rates, and eligibility for LDCT screening of patients diagnosed with lung cancer.

**RESULTS:**

From 2015 to May 2021, 22,886 patients were enrolled: 5,659 in LDCT, 15,461 in Lung Nodule, and 1,766 in Multidisciplinary Care. Of 150, 698, and 1,010 patients diagnosed with lung cancer in the respective programs, 61%, 60%, and 44% were diagnosed at clinical stage I or II, whereas 19%, 20%, and 29% were stage IV (*P* = .0005); 47%, 42%, and 32% had curative-intent surgery (*P* < .0001); aggregate 3-year overall survival rates were 80% (95% CI, 73 to 88) versus 64% (60 to 68) versus 49% (46 to 53); 5-year overall survival rates were 76% (67 to 87) versus 60% (56 to 65) versus 44% (40 to 48), respectively. Only 46% of 1,858 patients with lung cancer would have been deemed eligible for LDCT by US Preventive Services Task Force (USPSTF) 2013 criteria, and 54% by 2021 criteria. Even if all eligible patients by USPSTF 2021 criteria had been enrolled into LDCT, the Nodule Program would have detected 20% of the stage I-II lung cancer in the entire cohort.

**CONCLUSION:**

LDCT and Lung Nodule Programs are complementary, expanding access to early lung cancer detection and curative treatment to different-risk populations. Implementing Lung Nodule Programs may alleviate emerging disparities in access to early lung cancer detection.

## INTRODUCTION

Although aggregate US lung cancer incidence and mortality statistics have improved in recent years, they mask great geographic heterogeneity.^1-3^ States and counties in the Southeastern and Midwestern United States lag behind in the emerging improvement.^1,3^ The aggregate 5-year lung cancer survival barely reaches 21%, largely because 79% of patients present with regional and distant metastatic disease, when the 5-year survival is 32% and 6%, respectively.^1^ Only 15% present with localized disease when the 5-year survival is 59%.^1^

**CONTEXT**

**Key Objective**
How do the characteristics of patients enrolled into low-dose computed tomography lung cancer screening (LDCT) and Lung Nodule Programs compare with each other and with other patients managed in a Multidisciplinary Thoracic Oncology Program? Do these programs reach different-risk populations? How do the characteristics and outcomes of lung cancer diagnosed within each program compare?
**Knowledge Generated**
In this prospective observational cohort, we found that the proportion of early-stage diagnosis, curative-intent surgery, and overall survival were greater in patients in both types of early detection programs. However, most patients diagnosed with lung cancer in the Nodule Program would have been ineligible for LDCT by standard screening eligibility criteria.
**Relevance**
LDCT and Lung Nodule Programs have great complementarity in identifying early-stage lung cancer in diverse populations, when curative-intent treatment is more likely, safer, and less expensive. Implementing Lung Nodule Programs may expand access to early lung cancer detection, potentially reducing disparities.

Low-dose computed tomographic screening for lung cancer (LDCT) saves lives.^4,5^ Annual LDCT was recommended by the US Preventive Services Task Force (USPSTF) in 2013 for patients age 55-80 years with a 30 pack-year history of smoking within 15 years.^6^ The USPSTF extended eligibility in 2021 to individuals as young as 50 years, with the smoking intensity as low as 20 pack years, but retained the requirement for active smoking or cessation within 15 years.^7,8^ These criteria do not capture the full spectrum of individuals at risk for lung cancer.^8-11^ Screening rates in eligible US adults only increased from 3.3% in 2016 to 5% in 2018.^12^ Furthermore, eligibility for, access to, and participation in LDCT are rapidly expanding health care disparities.^11-15^ Current eligibility criteria underestimate the risk in women and racial minorities^11^; there is a geographic mismatch between per-capita lung cancer mortality rates and availability of lung cancer screening facilities.^12,15^

Irrespective of indication, radiologic tests often reveal potentially malignant lung lesions.^16^ Lung nodule management guidelines exist,^17-19^ but are infrequently followed.^20-22^ Guideline-concordant management of incidentally detected lung nodules requires infrastructure.^21,23^ The potential for synergy between such Lung Nodule and LDCT Programs is unclear, especially in high lung cancer incidence, granuloma-endemic regions such as the Mississippi Delta.

We compared individuals diagnosed with lung cancer through concurrently deployed LDCT and Lung Nodule (early detection) Programs and those managed in a Multidisciplinary Thoracic Oncology Program. We hypothesized that the characteristics of lung cancer would be similar between the early detection programs; they would have synergy by reaching different-risk populations; patients diagnosed through both programs would have earlier stage and better outcomes than those diagnosed outside them.

## METHODS

We constructed a prospective observational cohort, Detecting Early Lung Cancer (DELUGE) in the Mississippi Delta, using routinely generated clinical data of all patients managed through the LDCT or Lung Nodule Program with approval of the Baptist Memorial Health Care Corporation (BMHCC) Institutional Review Board. We compared DELUGE participants with participants in the Multidisciplinary Thoracic Oncology Program at BMHCC. BMHCC, a community-based health care system with institutions across Eastern Arkansas, Mississippi, and Western Tennessee, provides care to populations in 111 counties, including counties in Southwestern Kentucky, Southeastern Missouri, and Northwestern Alabama. These counties have some of the highest per-capita lung cancer incidence and mortality rates in the United States; 44% are persistent poverty counties in the Delta Regional Authority, whose 252 counties and parishes are designated by the US Congress as the most socioeconomically distressed.^24^

### Early Detection Programs

We implemented the *LDCT Program* in 2015 for consenting apparently healthy individuals who met USPSTF 2013 lung cancer screening eligibility criteria.^6^ We used the American College of Radiology Lung Imaging Reporting and Data System (Lung-RADS) to categorize patients' risk and triage care.^25,26^ Data from the LDCT program are prospectively reported to the American College of Radiology Registry.^25,27,28^

We concurrently implemented a *Lung Nodule Program* in 2015 as a safety net when radiologic studies, irrespective of indication (other than known or suspected cancer), revealed a potentially malignant lung lesion. Patients were automatically captured daily from radiology reports that included a standardized statement (Appendix, online only). Trained navigators used Fleischner Society Lung Nodule Management Guidelines for risk stratification.^17,19^

The provider who ordered the radiologic study was notified of the radiologist's concern and offered assistance with further management in the Nodule Program. Patients whose physicians accepted this offer were directly contacted and given the recommendations for subsequent care (additional radiologic or invasive testing, radiologic surveillance, or discharge). Patients for whom an invasive procedure was recommended were evaluated by a pulmonologist or general thoracic surgeon in a Lung Nodule Clinic and also presented in the Multidisciplinary Thoracic Oncology Conference. Patients whose physicians refused the offer of support were not contacted by the program staff.

### Multidisciplinary Thoracic Oncology Program

In 2011, we implemented a weekly Multidisciplinary Thoracic Oncology Conference involving thoracic surgeons, radiation oncologists, pulmonologists, medical oncologists, pathologists, radiologists, nurse navigators, and clinical research coordinators.^29,30^ Patients from within and outside the health care system with suspected lung cancer could be referred for discussion.

### Data Collection

Each program had its own specific, structured database. From 2011 onward, data on all patients presented in the Multidisciplinary Conference were captured in a database. From 2015 onward, information on patients with any radiologic test in which the standardized statement was used was captured in the Lung Nodule Program database; information on patients who underwent LDCT from across the health care system (identified through procedure code 71271) was entered into the LDCT database.

Trained data managers prospectively abstracted information from the electronic health record of all patients evaluated within each program. Data on clinical events were abstracted from routinely generated clinical records. Study data were collected and managed in research electronic data capture (REDCap), a secure, web-based software platform for research studies.^31,32^ Data abstraction, management, and updates followed prespecified standard operating procedures (Protocol, online only).

### Patient Selection and Categorization

For this analysis, we included patients in the LDCT or Nodule Program databases from 2015 to May 2021. The Multidisciplinary Care cohort included patients from 2015 to December 2020, who were not registered in either early detection database. Rurality was determined by the Rural-Urban Commuting Area code of the patient's zip code of residence at diagnosis.^33^

### Vital Statistics

Death information was obtained from prospective review of the electronic health record and from the institution's tumor registries at six-month intervals.

### Statistical Analysis

We summarized patient characteristics, care delivery and outcomes with frequencies, percentages, medians, and interquartile ranges (IQRs) and used chi-squared tests, Fisher's exact tests, and type III overall F tests from analysis of variance for comparisons across programs. We estimated overall survival using the Kaplan-Meier method and compared it across programs using the log-rank test. We further evaluated the survival impact of the two early detection programs using Cox proportional hazard regression to estimate pairwise hazard ratios, with 95% CI comparing each program. We used unadjusted models and multivariable models adjusted for age, sex, race, insurance, rurality, smoking status, and Charlson comorbidity index. Survival information was censored on August 31, 2021, and patients with missing mortality data were excluded. We set the alpha level at .05 and applied the false discovery rate approach to account for multiple comparisons.^34^ Statistical analyses were performed using R Studio 4.7 (Vienna, Austria) and SAS Version 9.4 (Cary, NC). We followed the Strengthening the Reporting of Observational Studies in Epidemiology reporting guidelines.

## RESULTS

### Characteristics of Patients in Each Program

**FIG 1.**
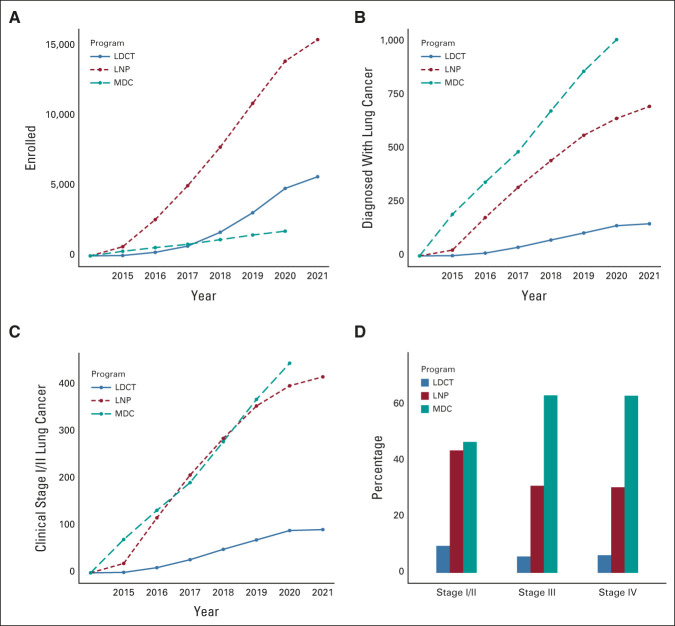
Cumulative enrollment of patients into LDCT, LNP, and MDC Programs: (A) patients enrolled, (B) patients diagnosed with lung cancer, and (C) patients diagnosed with clinical stage I or II lung cancer. (D) Proportions of the whole cohort of 1,858 patients diagnosed with lung cancer who had stage I/II, III, and IV lung cancer identified through each program (clinical stage distribution transcohort). LDCT, Low-Dose Computed Tomography Lung Cancer Screening Program; LNP, Lung Nodule Program; MDC, Multidisciplinary Care Program.

The cohort included 5,659 patients in LDCT, 15,461 in the Lung Nodule, and 1,766 in the Multidisciplinary Care Programs (Fig 1A and Table 1). Patients in the Nodule Program were younger, more likely to be female, least likely to be White, and most likely to be uninsured than patients in the other programs. Patients in the Multidisciplinary Program were most likely to have Medicaid insurance. The majority of LDCT enrollees were people who actively smoke (67%), whereas the majority in the Nodule and Multidisciplinary Programs had either quit (27% and 40%, respectively) or had never smoked (38% and 21%). Intensity of tobacco use, measured in pack years, was higher for patients in the LDCT than Nodule and Multidisciplinary Programs, irrespective of current smoking status (*P* < .0001). The quit duration was a median of 7 years (IQR: 4-11) in the LDCT, 17 years (7-32) in the Nodule, and 12 years (5-25) in the Multidisciplinary Program (*P* < .0001).

**TABLE 1.**
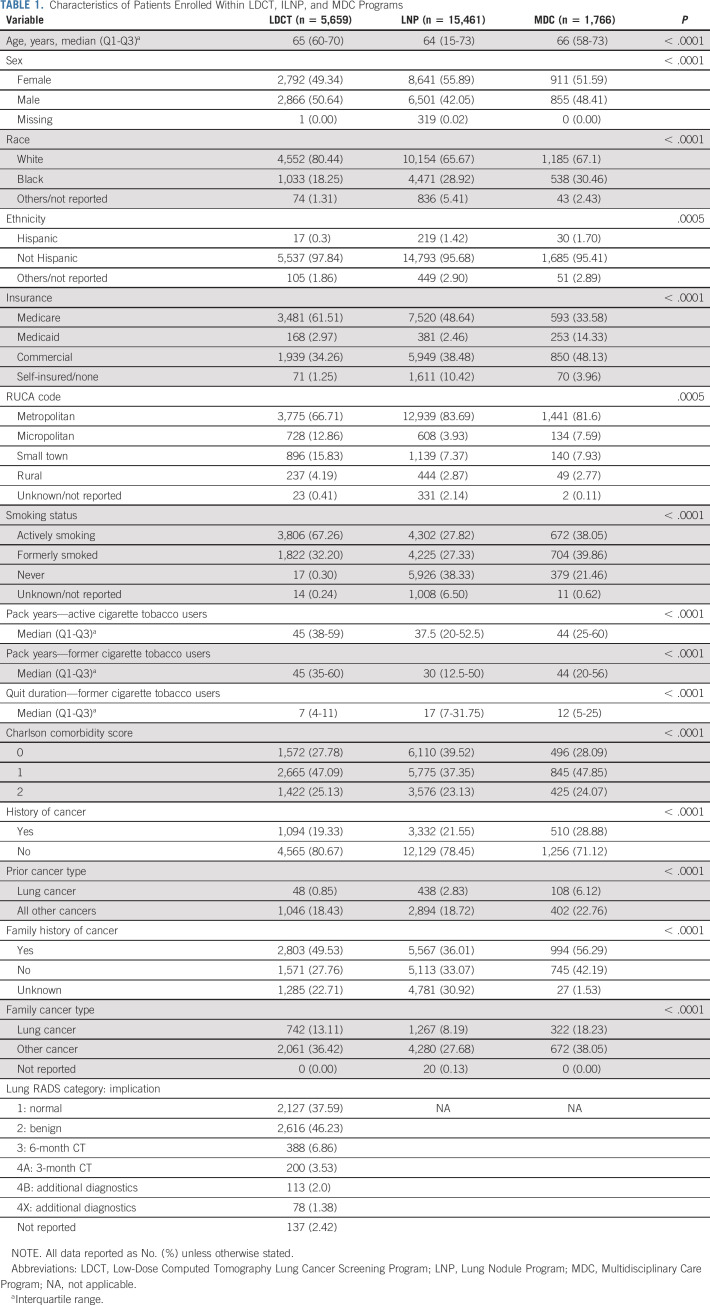
Characteristics of Patients Enrolled Within LDCT, ILNP, and MDC Programs

A substantial minority of patients in the LDCT, Lung Nodule, and Multidisciplinary Care cohorts had a history of cancer (19%, 22%, and 29%, respectively, *P* < .0001) although only 1%, 3%, and 6% had previously had lung cancer. A family history of lung cancer was identified in 13%, 8%, and 18%. In the LDCT cohort, 84% were initially categorized as Lung-RADS 1 or 2, 7% as 3, and 7% as 4 (Table 1). Restricting the Lung Nodule Program cohort to age 50-80 years did not substantively change the comparative characteristics (Appendix Table A1, online only).

### Characteristics of Patients Diagnosed With Cancer

Cancer was diagnosed in 156 of 5,659 (3%) patients in the LDCT, 772 of 15,461 (5%) in the Nodule, and 1,139 of 1,766 (65%), in the Multidisciplinary Programs (Table 2). Compared with patients in the LDCT program, patients diagnosed with cancer from the Nodule Program were more likely to be Black (27% *v* 16%) or uninsured (4% *v* 1%). They were also more likely to have quit smoking (40% *v* 28%); in addition, 13% had never smoked. Similarly, 14% of patients diagnosed with cancer in the Multidisciplinary Program had never smoked. By contrast, 72% of LDCT enrollees diagnosed with cancer still actively smoked.

**TABLE 2.**
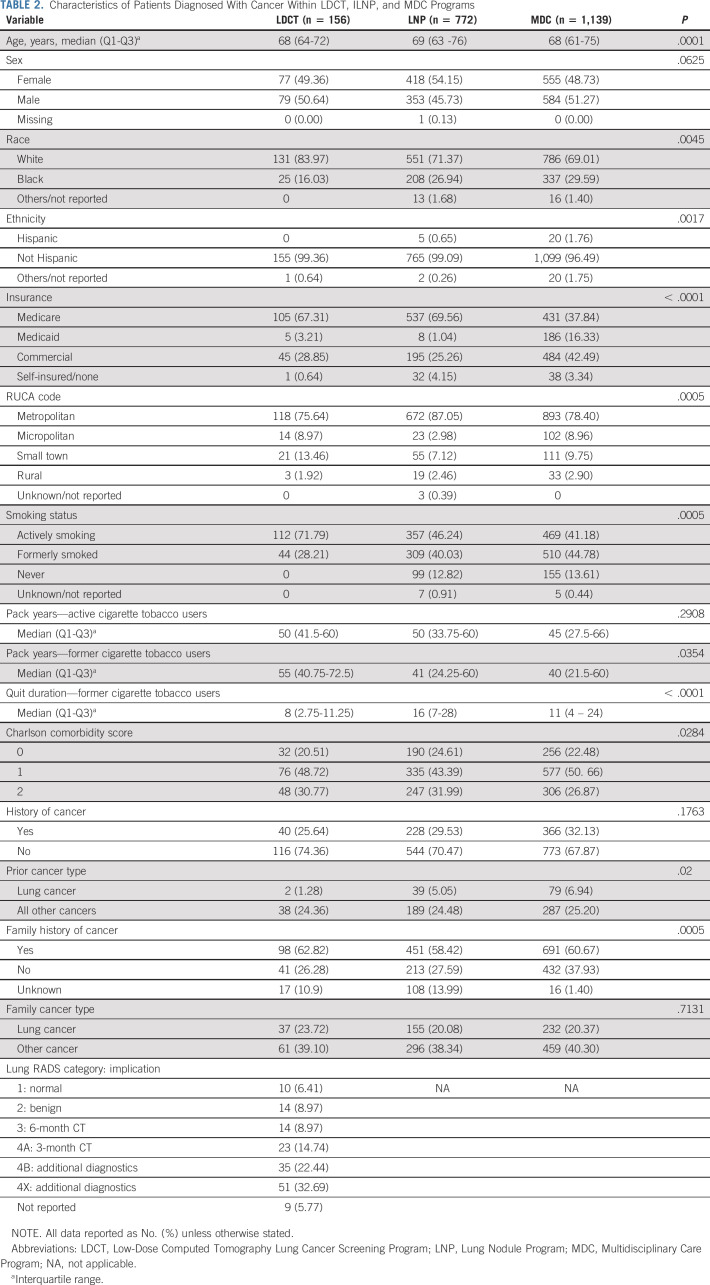
Characteristics of Patients Diagnosed With Cancer Within LDCT, ILNP, and MDC Programs

The median quit duration was 8 (IQR: 3-11) years in the LDCT, 16 (7-28) years in the Nodule Program, and 11 (4-24) years in the Multidisciplinary Program cohort diagnosed with cancer (*P* < .0001). Similar proportions of patients in the three programs had a personal history of cancer: 26% versus 30% versus 32% (*P* = .1763); but LDCT enrollees were least likely to have previously had lung cancer—1% versus 5% versus 7%, respectively (*P* = .020). Twenty-four percent, 20%, and 20% of patients diagnosed with cancer from each respective program had a family history of lung cancer. The first Lung-RADS scores of LDCT enrollees were 1 in 6%, 2 in 9%, 3 in 9%, and 4 in 70%. Results were similar in the Lung Nodule Program cohort age 50-80 years (Appendix Table A2, online only).

### Lung Cancer Characteristics and Treatment Across Programs

**FIG A1.**
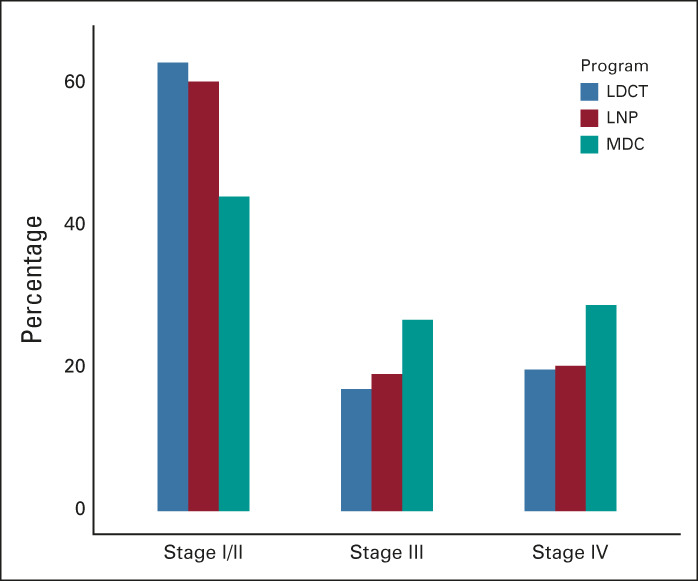
Stage distribution of lung cancer patients diagnosed through three programs. LDCT, Low-Dose Computed Tomography Lung Cancer Screening Program; LNP, Lung Nodule Program; MDC, Multidisciplinary Care Program.

One hundred fifty, 698, and 1,010 patients had lung cancer (Fig 1B): adenocarcinoma was the most common histology—52% versus 48% versus 42%; 12%, 8%, and 8% had small-cell lung cancer; but 4%, 10%, and 11% did not have primary lung cancer (Appendix Table A3, online only). The stage distribution was I or II in 61% versus 60% versus 44%, respectively, and stage IV in 19%, 20%, and 29% (*P* = .0005; Appendix Table A4, Appendix Fig A1, online only). Of 955 patients diagnosed at stage I or II, 92 (10%) were enrolled in LDCT, 417 (44%) in the Nodule, and 446 (47%) in the Multidisciplinary Program (Figs 1C and 1D). The median primary tumor size was 19.5 mm (IQR: 13-30), 25 mm (16-41), and 35 mm (21-53), respectively (*P* < .0001).

**FIG 2.**
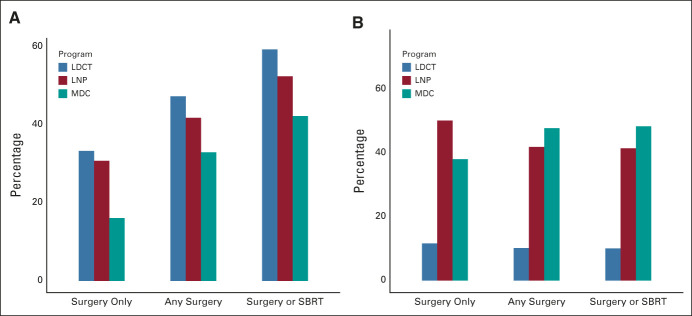
Use of surgery only, surgery with or without other treatment modality (any surgery), and surgery or stereotactic radiosurgery (surgery or SBRT) to treat patients diagnosed with lung cancer through LDCT, LNP, and MDC Programs: (A) proportions within each program (denominator is patients diagnosed with lung cancer within each program) and (B) proportions of the whole cohort (denominator is all 1,858 patients diagnosed with lung cancer from all three programs combined). LDCT, Low-Dose Computed Tomography Lung Cancer Screening Program; LNP, Lung Nodule Program; MDC, Multidisciplinary Care Program; SBRT, stereotactic body radiotherapy.

Lung cancer treatment included surgery in 47% of patients in LDCT, 42% in Nodule, and 33% in the Multidisciplinary Program, including surgery without adjuvant therapy in 33%, 31%, and 16%, respectively (*P* < .0001; Fig 2A). However, of the 696 patients who underwent surgical resection, 10% were from the LDCT, 42% from the Nodule Program, and 48% from the Multidisciplinary Program (Fig 2B). Of the 428 patients who had surgery alone, 12% were from the LDCT, 50% were from the Nodule, and 38% were from the Multidisciplinary Program; 2%, 3%, and 9% of patients with lung cancer in the respective programs received no treatment.

### Comparative Lung Cancer Outcomes

**FIG 3.**
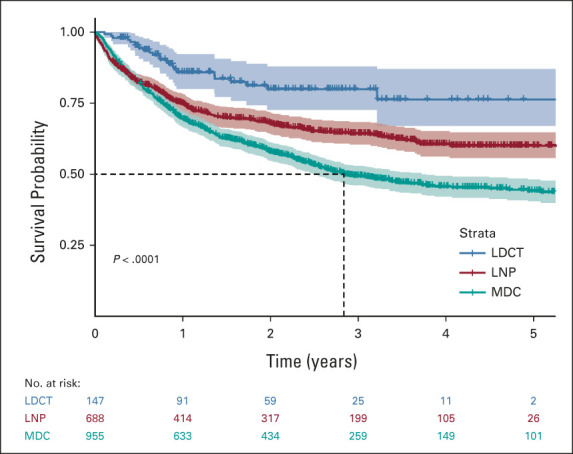
Kaplan-Meier survival plots of patients diagnosed with lung cancer who were enrolled into LDCT, LNP, and MDC Programs. LDCT, Low-Dose Computed Tomography Lung Cancer Screening Program; LNP, Lung Nodule Program; MDC, Multidisciplinary Care Program.

The postoperative mortality at 120 days was 0% in the LDCT, 4% in the Nodule, and 8% in the Multidisciplinary Care Program (*P* = .0053, Appendix Table A3). Aggregate crude 3-year survival rates were 80% (95% CI, 73 to 88), 64% (60 to 68), and 49% (46 to 53); 5-year survival rates were 76% (95% CI, 67 to 87), 60% (56 to 65), and 44% (40 to 48), respectively (log-rank *P* < .0001; Fig 3).

The overall hazard of death was lower in patients from the LDCT and Nodule Programs compared with the Multidisciplinary Program. After adjustment for age, sex, race, insurance, patient-level rurality, smoking status, and comorbidities, patients from the LDCT Program had an aggregate HR of 0.39 (95% CI, 0.228 to 0.65) and those from the Nodule Program had 0.74 (0.59 to 0.921) compared with the Multidisciplinary Program (Appendix Table A4). On aggregate, LDCT patients with lung cancer had better survival than Lung Nodule Program patients (HR, 0.46; 95% CI, 0.298 to 0.704).

### Eligibility for LDCT Lung Cancer Screening Among Patients Diagnosed With Lung Cancer

By USPSTF 2013 criteria, 89%, 43%, and 43% of patients with lung cancer diagnosed through the LDCT, Lung Nodule, and Multidisciplinary Programs were eligible for LDCT screening (Table 3). By the USPSTF's revised criteria proposed in 2021, 91%, 49%, and 52% would have been eligible. Framed differently, only 861 (46%) and 1,010 (54%) of a total of 1,858 patients with lung cancer in this cohort would have been deemed eligible for LDCT lung cancer screening by USPSTF 2013 and 2021 criteria. Results were similar in the Nodule Program cohort age 50-80 years (Appendix Table A5, online only). Even if all eligible patients by USPSTF 2021 criteria had been enrolled into the LDCT program, the Nodule Program would have detected 189 (20%) of the 955 patients with stage I and II lung cancer in the entire cohort.

**TABLE 3.**
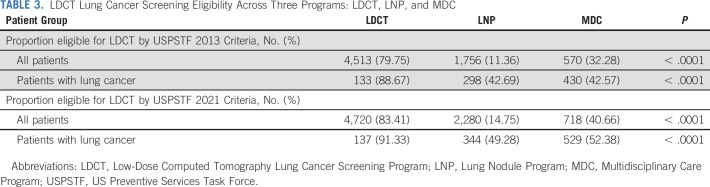
LDCT Lung Cancer Screening Eligibility Across Three Programs: LDCT, LNP, and MDC

## DISCUSSION

In this cohort from two early lung cancer detection programs in a large community health care system, 2.7 patients were enrolled into the Lung Nodule Program for every one in LDCT, five patients were diagnosed with lung cancer in the Nodule Program for every one patient diagnosed in LDCT, and 4.5 patients were diagnosed with stage I or II lung cancer for every one in LDCT. Stage distribution was similar in both early detection programs and significantly earlier than in the Multidisciplinary Program. Consequently, the proportion of curative-intent surgery was greater in patients in the early detection programs. However, most patients diagnosed with lung cancer in the Nodule Program would have been ineligible for LDCT screening by then-current, and recently proposed, USPSTF criteria.

Surgery was safe for patients with LDCT-detected lung cancer in this community health care program; the postoperative mortality at 120 days was zero. Aggregate survival was better in the patients enrolled into both early detection programs; the LDCT cohort had better aggregate survival than the Lung Nodule Cohort. This difference between early detection programs might be because LDCT eligibility requires ostensible good health, whereas the Lung Nodule Program patients had a myriad of clinical indications for the lesion-detecting radiologic study.

Even if all eligible patients were enrolled into LDCT, 46% of patients diagnosed with lung cancer would have been deemed ineligible. In reality, given the prevailing low levels of adoption, only 8% of patients with lung cancer in this cohort received LDCT screening; 38% were detected through the Nodule Program, including 44% of patients diagnosed with localized disease (*v* 10% by LDCT). This reveals great complementarity between LDCT and Lung Nodule Programs, tandem deployment of which can extend the possibility of early detection of lung cancer to a wider spectrum of the population than LDCT alone. The programs reached different demographic segments of the population, potentially providing a means of overcoming the looming problem of access disparity to lung cancer screening, which threatens to widen racial, sex, and socioeconomic and geographic disparities in lung cancer mortality.^11-15,35-42^

Despite evidence from two large randomized clinical trials that lung cancer screening saves lives, adoption has been slow, difficult, and complex, even in the United States where social policy moved in 2015 to provide payment for annual LDCT screening as part of standard health care coverage.^6,43^ Barriers to implementation include the need for infrastructure for recruiting candidates who meet eligibility criteria for screening and conducting the mandated shared decision making and tobacco cessation counseling services required before the LDCT screening test can be performed.^27,28^ Organizations that manage high-risk patients and primary care providers who are the main entry portal into LDCT screening programs can feel overburdened by these requirements.^44-47^ Opening access to the indigent and other hard-to-reach populations at high risk for lung cancer is a major challenge.

These barriers diminish the societal benefit of LDCT screening, and some may be alleviated by structured implementation of Lung Nodule programs, which start from the point of an already-detected radiologic lesion and triage patients into risk categories for subsequent management. Institutional investment in Nodule Program infrastructure may be easier to justify for medicolegal risk management. Implementation of Lung Nodule Programs ought to be possible in environments where there is no policy support for LDCT screening.^48^

Prospective observational studies are open to selection and misclassification biases. LDCT enrollees are ostensibly healthier by eligibility criteria; given the striking differences in the smoking profile, the genetic profile of the lung cancers diagnosed through the different programs might differ; care for patients in the Nodule Program might be adversely affected by the need to manage the initial indication for radiologic imaging that led to detection of the lung lesion. Furthermore, the Multidisciplinary Care cohort is not the optimal comparator for early detection programs, given the referral bias into multidisciplinary care programs. We have previously demonstrated superior care and survival for patients enrolled into this Multidisciplinary Care Program when compared with the general population of patients with lung cancer within the same health care system.^30^ Moreover, some Multidisciplinary Program patients might have had an LDCT scan or been managed for an incidentally detected lung nodule outside our program.

The possibility of selection bias is supported by the higher proportion of early stage (44%) and lower-than-expected proportion of patients with stage IV (29%) as compared with the US distribution (15% early stage and 40%-50% stage IV).^1^ These features may bias the survival comparison toward the null, indicating that the true impact of tandem deployment of the early detection programs is probably significantly greater than that we report. Survival improvement from early cancer detection raises questions about lead-time and overdiagnosis bias, which can be lessened by analysis of mortality events. We plan to evaluate cause-specific mortality in future studies. Finally, we have not separated between incident and prevalence lung cancers in this analysis. The fact that 24% of patients with lung cancer in the LDCT cohort had Lung-RADS 1-3 in their initial scans emphasizes the need to ensure adherence to the recommended follow-up testing regimen.

LDCT and Lung Nodule Programs have great complementarity in redistributing lung cancer to early stage when curative-intent treatment is more likely, safer, and less expensive. Lung Nodule Programs may expand access to hard-to-reach individuals and may be less susceptible to multilevel implementation barriers.

## Figures and Tables

**TABLE A1.**
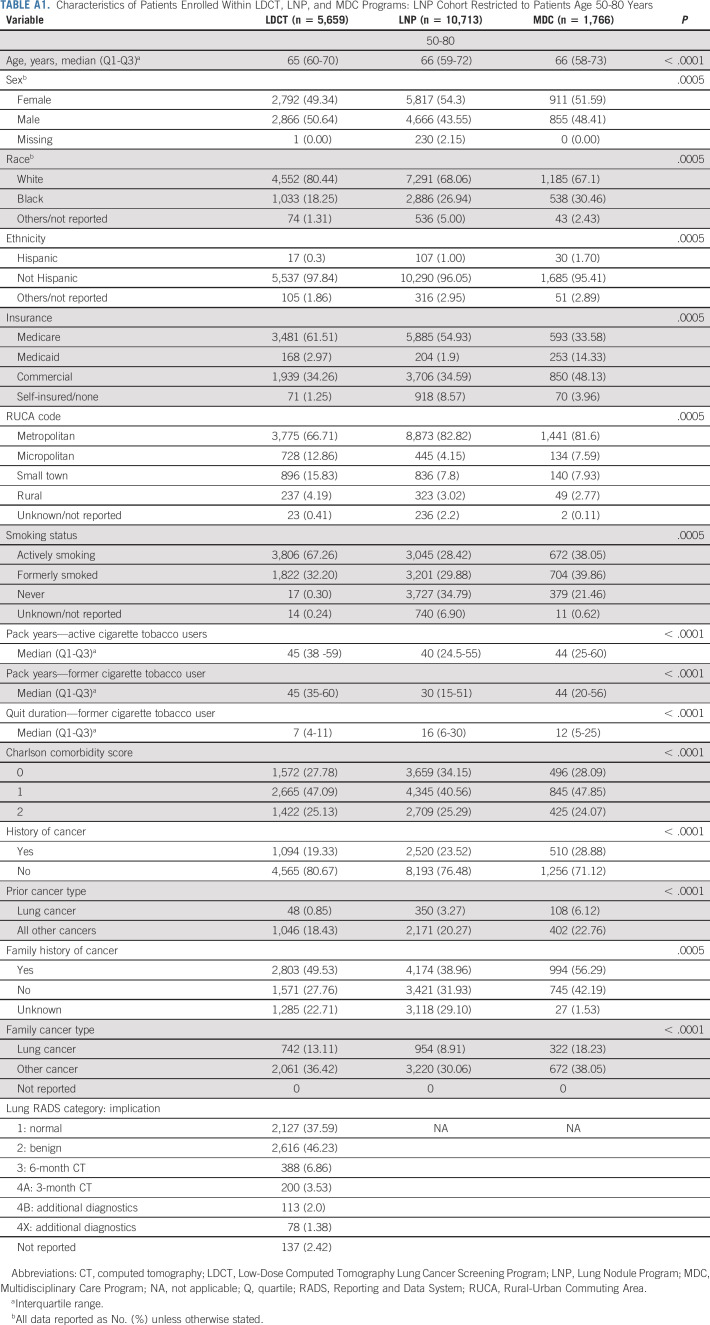
Characteristics of Patients Enrolled Within LDCT, LNP, and MDC Programs: LNP Cohort Restricted to Patients Age 50-80 Years

**TABLE A2.**
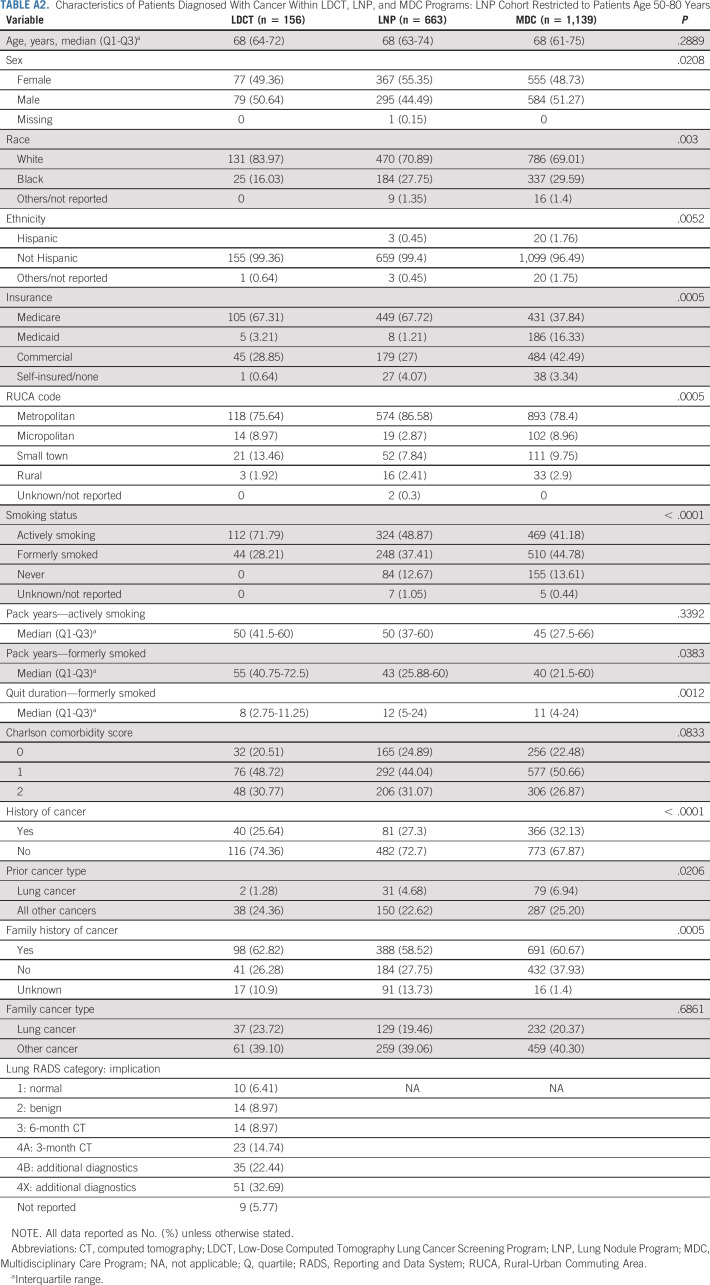
Characteristics of Patients Diagnosed With Cancer Within LDCT, LNP, and MDC Programs: LNP Cohort Restricted to Patients Age 50-80 Years

**TABLE A3.**
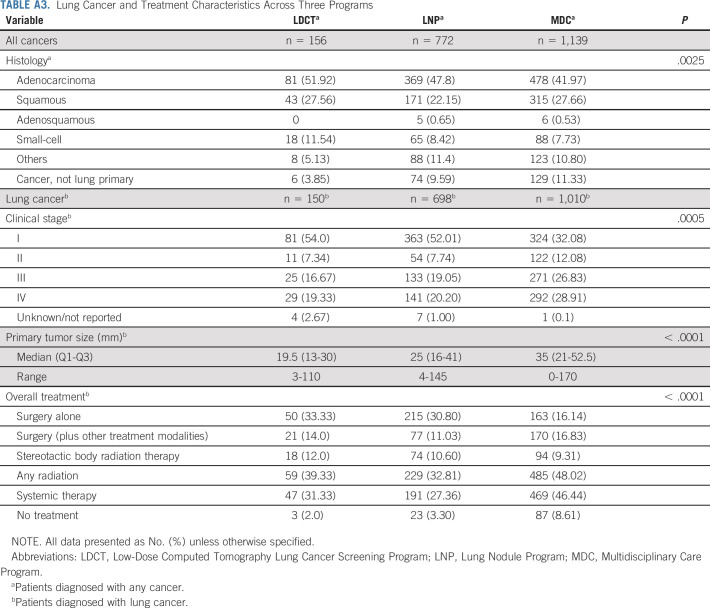
Lung Cancer and Treatment Characteristics Across Three Programs

**TABLE A4.**
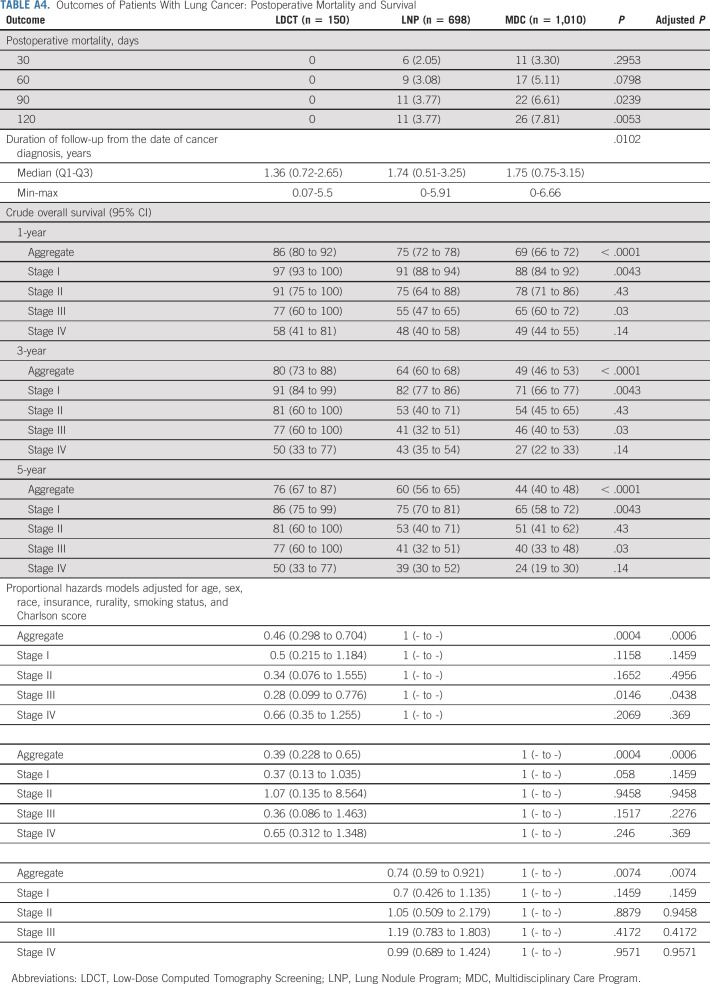
Outcomes of Patients With Lung Cancer: Postoperative Mortality and Survival

**TABLE A5.**
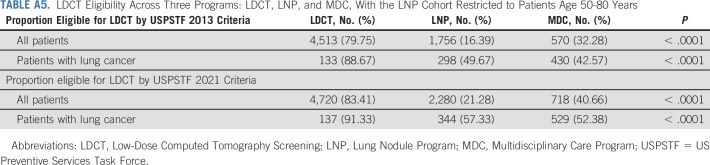
LDCT Eligibility Across Three Programs: LDCT, LNP, and MDC, With the LNP Cohort Restricted to Patients Age 50-80 Years

## APPENDIX

Standard radiology statement from which automated capture of radiology reports is placed into a daily queue for review by the Lung Nodule Program navigators: “Certain findings detected within the thorax (chest) on this exam warrant follow up, as per published guidelines. Additional evaluation of these findings is recommended.”

## References

[1] Siegel RL Miller KD Fuchs HE : Cancer statistics, 2021. CA Cancer J Clin 71:7-33, 2021 [Erratum: CA Cancer J Clin 71:359, 2021] 33433946 10.3322/caac.21654

[2] Howlader N Forjaz G Mooradian MJ : The effect of advances in lung-cancer treatment on population mortality. N Engl J Med 383:640-649, 2020 32786189 10.1056/NEJMoa1916623 PMC8577315

[3] Mokdad AH Dwyer-Lindgren L Fitzmaurice C : Trends and patterns of disparities in cancer mortality among US counties, 1980-2014. JAMA 317:388-406, 2017 28118455 10.1001/jama.2016.20324 PMC5617139

[4] National Lung Screening Trial Research Team, Aberle DR Adams AM : Reduced lung-cancer mortality with low-dose computed tomographic screening. N Engl J Med 365:395-409, 2011 21714641 10.1056/NEJMoa1102873 PMC4356534

[5] de Koning HJ van der Aalst CM de Jong PA : Reduced lung-cancer mortality with volume CT screening in a randomized trial. N Engl J Med 382:503-513, 2020 31995683 10.1056/NEJMoa1911793

[6] Moyer VA, US Preventive Services Task Force: Screening for lung cancer: U.S. Preventive Services Task Force recommendation statement. Ann Intern Med 160:330-338, 2014 24378917 10.7326/M13-2771

[7] US Preventive Services Task Force, Krist AH Davidson KW : Screening for lung cancer: US Preventive Services Task Force recommendation statement. JAMA 325:962-970, 2021 33687470 10.1001/jama.2021.1117

[8] Meza R Jeon J Toumazis I : Evaluation of the benefits and harms of lung cancer screening with low-dose computed tomography: Modeling study for the US Preventive Services Task Force. JAMA 325:988-997, 2021 33687469 10.1001/jama.2021.1077 PMC9208912

[9] Han SS Chow E Ten Haaf K : Disparities of National Lung Cancer Screening guidelines in the US population. J Natl Cancer Inst 112:1136-1142, 2020 32040195 10.1093/jnci/djaa013 PMC7669226

[10] Landy R Young CD Skarzynski M : Using prediction-models to reduce persistent racial/ethnic disparities in draft 2020 USPSTF lung-cancer screening guidelines. J Natl Cancer Inst 113:1590-1594, 2021 33399825 10.1093/jnci/djaa211 PMC8562965

[11] Pinsky PF Lau YK Doubeni CA: Potential disparities by sex and race or ethnicity in lung cancer screening eligibility rates. Chest 160:341-350, 2021 33545164 10.1016/j.chest.2021.01.070 PMC8411441

[12] Fedewa SA Kazerooni EA Studts JL : State variation in low-dose computed tomography scanning for lung cancer screening in the United States. J Natl Cancer Inst 113:1044-1052, 2021 33176362 10.1093/jnci/djaa170 PMC8328984

[13] Zahnd WE Eberth JM: Lung cancer screening utilization: A behavioral risk factor surveillance system analysis. Am J Prev Med 57:250-255, 2019 31248742 10.1016/j.amepre.2019.03.015

[14] Aldrich MC Mercaldo SF Sandler KL : Evaluation of USPSTF lung cancer screening guidelines among African American adult smokers. JAMA Oncol 5:1318-1324, 2019 31246249 10.1001/jamaoncol.2019.1402 PMC6604090

[15] Sahar L Douangchai Wills VL Liu KK : Using geospatial analysis to evaluate access to lung cancer screening in the United States. Chest 159:833-844, 2021 32888933 10.1016/j.chest.2020.08.2081

[16] Gould MK Tang T Liu IL : Recent trends in the identification of incidental pulmonary nodules. Am J Respir Crit Care Med 192:1208-1214, 2015 26214244 10.1164/rccm.201505-0990OC

[17] MacMahon H Austin JH Gamsu G : Guidelines for management of small pulmonary nodules detected on CT scans: A statement from the Fleischner Society. Radiology 237:395-400, 2005 16244247 10.1148/radiol.2372041887

[18] Gould MK Donington J Lynch WR : Evaluation of individuals with pulmonary nodules: When is it lung cancer? Diagnosis and management of lung cancer, 3rd ed: American College of Chest Physicians evidence-based clinical practice guidelines. Chest 143:e93S-e120S, 2013 (5 suppl) 23649456 10.1378/chest.12-2351 PMC3749714

[19] MacMahon H Naidich DP Goo JM : Guidelines for management of incidental pulmonary nodules detected on CT images: From the Fleischner Society. Radiology 284:228-243, 2017 28240562 10.1148/radiol.2017161659

[20] Vachani A Tanner NT Aggarwal J : Factors that influence physician decision making for indeterminate pulmonary nodules. Ann Am Thorac Soc 11:1586-1591, 2014 25386795 10.1513/AnnalsATS.201405-197BC PMC5475427

[21] Wiener RS Gould MK Slatore CG : Resource use and guideline concordance in evaluation of pulmonary nodules for cancer: Too much and too little care. JAMA Intern Med 174:871-880, 2014 24710850 10.1001/jamainternmed.2014.561 PMC4266552

[22] Tanner NT Aggarwal J Gould MK : Management of pulmonary nodules by community pulmonologists: A multicenter observational study. Chest 148:1405-1414, 2015 26087071 10.1378/chest.15-0630 PMC4665735

[23] Wiener RS Slatore CG Gillespie C : Pulmonologists' reported use of guidelines and shared decision-making in evaluation of pulmonary nodules: A qualitative study. Chest 148:1415-1421, 2015 25789979 10.1378/chest.14-2941 PMC4665736

[24] About | Delta Regional Authority. dra.gov

[25] Kazerooni EA Armstrong MR Amorosa JK : ACR CT accreditation program and the lung cancer screening program designation. J Am Coll Radiol 12:38-42, 2015 25455196 10.1016/j.jacr.2014.10.002

[26] Pinsky PF Gierada DS Black W : Performance of Lung-RADS in the National Lung Screening Trial: A retrospective assessment. Ann Intern Med 162:485-491, 2015 25664444 10.7326/M14-2086 PMC4705835

[27] Mazzone P Powell CA Arenberg D : Components necessary for high-quality lung cancer screening: American College of Chest Physicians and American Thoracic Society policy statement. Chest 147:295-303, 2015 25356819 10.1378/chest.14-2500 PMC4502754

[28] Fintelmann FJ Bernheim A Digumarthy SR : The 10 pillars of lung cancer screening: Rationale and logistics of a lung cancer screening program. Radiographics 35:1893-1908, 2015 26495797 10.1148/rg.2015150079

[29] Smeltzer MP Rugless FE Jackson BM : Pragmatic trial of a multidisciplinary lung cancer care model in a community healthcare setting: Study design, implementation evaluation, and baseline clinical results. Transl Lung Cancer Res 7:88-102, 2018 29535915 10.21037/tlcr.2018.01.02 PMC5835591

[30] Ray MA Faris NR Fehnel C : Survival impact of an enhanced multidisciplinary thoracic oncology conference in a regional community health care system. JTO Clin Res Rep 2:100203, 2021 34590046 10.1016/j.jtocrr.2021.100203 PMC8474211

[31] Harris PA Taylor R Thielke R : Research electronic data capture (REDCap)—A metadata-driven methodology and workflow process for providing translational research informatics support. J Biomed Inform 42:377-381, 2009 18929686 10.1016/j.jbi.2008.08.010 PMC2700030

[32] Harris PA Taylor R Minor BL : The REDCap consortium: Building an international community of software partners. J Biomed Inform 95:103208, 2019 31078660 10.1016/j.jbi.2019.103208 PMC7254481

[33] https://www.ers.usda.gov/data-products/rural-urban-commuting-area-codes.aspx

[34] Benjamini Y Hochberg Y: Controlling the false discovery rate: A practical and powerful approach to multiple testing. J R Stat Soc Ser B 57:289-300, 1995

[35] Lake M Shusted CS Juon HS : Black patients referred to a lung cancer screening program experience lower rates of screening and longer time to follow-up. BMC Cancer 20:561, 2020 32546140 10.1186/s12885-020-06923-0 PMC7298866

[36] Rivera MP Katki HA Tanner NT : Addressing disparities in lung cancer screening eligibility and healthcare access. An official American Thoracic Society statement. Am J Respir Crit Care Med 202:e95-e112, 2020 33000953 10.1164/rccm.202008-3053ST PMC7528802

[37] Reese TJ Schlechter CR Potter LN : Evaluation of revised US Preventive Services Task Force lung cancer screening guideline among women and racial/ethnic minority populations. JAMA Netw Open 4:e2033769, 2021 33433600 10.1001/jamanetworkopen.2020.33769 PMC7804914

[38] Shusted CS Evans NR Juon HS : Association of race with lung cancer risk among adults undergoing lung cancer screening. JAMA Netw Open 4:e214509, 2021 33822072 10.1001/jamanetworkopen.2021.4509 PMC8025120

[39] Van Hal G Diab Garcia P: Lung cancer screening: Targeting the hard to reach-a review. Transl Lung Cancer Res 10:2309-2322, 2021 34164279 10.21037/tlcr-20-525 PMC8182716

[40] Núñez ER Caverly TJ Zhang S : Adherence to follow-up testing recommendations in US veterans screened for lung cancer, 2015-2019. JAMA Netw Open 4:e2116233, 2021 34236409 10.1001/jamanetworkopen.2021.16233 PMC8267608

[41] Boudreau JH Miller DR Qian S : Access to lung cancer screening in the Veterans Health Administration: Does geographic distribution match need in the population? Chest 160:358-367, 2021 33617804 10.1016/j.chest.2021.02.016 PMC8640836

[42] Kunitomo Y Bade B Gunderson CG : Racial differences in adherence to lung cancer screening follow-up: A systematic review and meta-analysis. Chest 161:266-275, 2022 34390706 10.1016/j.chest.2021.07.2172

[43] Black WC Gareen IF Soneji SS : Cost-effectiveness of CT screening in the National Lung Screening Trial. N Engl J Med 371:1793-1802, 2014 25372087 10.1056/NEJMoa1312547 PMC4335305

[44] Seehusen DA: Should family physicians routinely screen for lung cancer in high-risk populations? No: The USPSTF's recommendation for lung cancer screening is overreaching. Am Fam Physician 90:73D-4D, 2014 25077590

[45] Wiener RS Gould MK Arenberg DA : ATS/ACCP Committee on Low-Dose CT Lung Cancer Screening in clinical practice. An official American Thoracic Society/American College of Chest Physicians policy statement: Implementation of low-dose computed tomography lung cancer screening programs in clinical practice. Am J Respir Crit Care Med 192:881-891, 2015 26426785 10.1164/rccm.201508-1671ST PMC4613898

[46] Kinsinger LS Anderson C Kim J : Implementation of lung cancer screening in the Veterans Health Administration. JAMA Intern Med 177:399-406, 2017 28135352 10.1001/jamainternmed.2016.9022

[47] Blom EF Ten Haaf K Arenberg DA : Treatment capacity required for full-scale implementation of lung cancer screening in the United States. Cancer 125:2039-2048, 2019 30811590 10.1002/cncr.32026 PMC6541509

[48] Field JK deKoning H Oudkerk M : Implementation of lung cancer screening in Europe: Challenges and potential solutions: Summary of a multidisciplinary roundtable discussion. ESMO Open 4:e000577, 2019 31673428 10.1136/esmoopen-2019-000577 PMC6802961

